# Human Parvovirus B19 NS1 Protein Aggravates Liver Injury in NZB/W F1 Mice

**DOI:** 10.1371/journal.pone.0059724

**Published:** 2013-03-21

**Authors:** Chun-Chou Tsai, Chun-Ching Chiu, Jeng-Dong Hsu, Huai-Sheng Hsu, Bor-Show Tzang, Tsai-Ching Hsu

**Affiliations:** 1 Institute of Microbiology and Immunology, Chung Shan Medical University, Taichung, Taiwan; 2 Department of Neurology and Department of Medical Intensive Care Unit, Chunghua Christian Hospital, Chunghua, Taiwan; 3 Department of Pathology, School of Medicine, Chung Shan Medical University, Taichung, Taiwan; 4 Department of Pathology, Chung Shan Medical University Hospital, Taichung, Taiwan; 5 Department of Biochemistry, School of Medicine, Chung Shan Medical University, Taichung, Taiwan; 6 Institute of Biochemistry and Biotechnology, Chung Shan Medical University, Taichung, Taiwan; 7 Clinical Laboratory, Chung Shan Medical University Hospital, Taichung, Taiwan; National Institute of Dental and Craniofacial Research, United States of America

## Abstract

Human parvovirus B19 (B19) has been associated with a variety of diseases. However, the influence of B19 viral proteins on hepatic injury in SLE is still obscure. To elucidate the effects of B19 viral proteins on livers in SLE, recombinant B19 NS1, VP1u or VP2 proteins were injected subcutaneously into NZB/W F1 mice, respectively. Significant expressions of inducible nitric oxide synthase (iNOS) and cyclooxygenase-2 (COX-2) were detected in NZB/W F1 mice receiving B19 NS1 as compared to those mice receiving PBS. Markedly hepatocyte disarray and lymphocyte infiltration were observed in livers from NZB/WF 1 mice receiving B19 NS1 as compared to those mice receiving PBS. Additionally, significant increases of Tumor Necrosis Factor –α (TNF-α), TNF-α receptor, IκB kinase –α (IKK-α), nuclear factor of kappa light polypeptide gene enhancer in B-cells inhibitor (IκB) and nuclear factor-kappa B (NF-κB) were detected in livers from NZB/W F1 mice receiving B19 NS1 as compared to those mice receiving PBS. Accordingly, significant increases of matrix metalloproteinase-9 (MMP9) and U-plasminogen activator (uPA) were also detected in livers from NZB/W F1 mice receiving B19 NS1 as compared to those mice receiving PBS. Contrarily, no significant variation on livers from NZB/W F1 mice receiving B19 VP1u or VP2 was observed as compared to those mice receiving PBS. These findings firstly demonstrated the aggravated effects of B19 NS1 but not VP1u or VP2 protein on hepatic injury and provide a clue in understanding the role of B19 NS1 on hepatic injury in SLE.

## Introduction

Systematic lupus erythematosus (SLE) is known as a systemic autoimmune disorder that affects various organs including liver [1]. Various reports have indicated that growing population with liver disease was found in patients with SLE [2]–[4]. Although the occurrence of liver disease is not routinely screened, the incidence of hepatic abnormality in patients with SLE was reported as varying from 12% to 55% [4].

Human parvovirus B19 (B19) is known as an important human pathogen, which consists a nonstructural protein (NS1) and two capsid proteins, VP1 and VP2 [5]. Notably, B19 infection has been associated with a wide range of different pathologies and clinical manifestations including erythema infectiosum, arthropathy, thrombocytopenia, neurologic disorders, hepatitis, cardiovasculitis and autoimmune disorders [5]–[8]. Indeed, various studies have postulated a connection between B19 infection and liver injury. A clinical study reported the existence of B19 DNA in a liver biopsy specimen from a patient with acute hepatitis [9]. Another studies also suggested an important role of B19 infection in acute icteric hepatitis liver injury [10] and acute fulminant hepatitis with bone marrow failure [11]. In addition, B19 infection has been recognized to trigger the acute liver failure in a patient with Wilson disease [12].

Although there is no direct evidence of B19 virus in inducing autoimmune diseases, the association between B19 virus and pathogenesis of autoimmunity has been strongly suggested. Recently, human parvovirus B19 has been associated with SLE [6], [13]–[16]. However, little is known about the influence of B19 viral proteins on liver injury in SLE. In the present study, various recombinant B19 viral proteins were prepared and injected subcutaneously into NZB/W F1 mice to elucidate the effects of B19 viral proteins on livers in SLE.

## Materials and Methods

### Ethics

Animal experiments were approved by the Institutional Animal Care and Use Committee at Chung Shan Medical University.

### Preparation of Recombinant B19 Viral Proteins

The recombinant human parvovirus B19 proteins were prepared as descried elsewhere [17]–[19]. Briefly, the plasmid pQE40-NS1 containing nonstructural (NS1) gene of human parvovirus B19 was kindly provided by Professor Susanne Modrow, Institute for Medical Microbiology, Universität Regensburg, Regensburg, Germany. The NS1 protein was purified using Profinia denaturing IMAC purification kits and the Profinia protein purification system (Bio-Rad Laboratories, Inc. USA) according to the manufacturer's instructions [17]. The cDNA of B19 VP1u were constructed onto pET-32a plasmid and transformed into E. coli (BL21-DE3). The recombinant B19 VP1u protein were then purified by Ni-NTA spin column (Qiagen, Chatsworth, CA) and spun through P50 and P30 Amicon (Millipore Billerica, MA) to avoid contaminative and degraded proteins [18]. The purified recombinant B19 NS1 and VP1u proteins were also analyzed by HPLC and the purities the three purified recombinant proteins were over 98%. The VP2 open reading frame (ORF) was obtained from the B19 genome (plasmid pYT104-C) by polymerase chain reaction using primers 5′-CGGAATTCCATGACTTCAGTTAATTCTGCAGAAGCC-3′ and 5′-GCGCGG CCGCTTACAATGGGTGCACACGGC-3′ containing *EcoR I* and *Not I* recognition sequences for subsequent cloning to pVL1393 baculoviral transfer vectors (Invitrogen). The constructed transfer vector and the BaculoGold DNA were used to co-transfect *Spodoptera frugiperda* (Sf9) cells by the calcium phosphate coprecipitation method according to the protocol provided by the manufacturer (PharMingen, San Diego, CA). Sf9 cells (Novagen, Merck, Germany) were maintained in Sf-900 II SFM (Invitrogen) in 100% room air at 28°C. Sf9 cells were infected with baculovirus stocks at a multiplicity of infection (MOI) of 5 and were harvested 72 h after infection. Recombinant VP2 proteins were purified by using 20% sucrose cushion and CsCl density gradient as described else where [19]. The yields of purified recombinant B19-NS1, -VP1u and VP2 proteins are 6.1, 15.6 and 17.7 mg/l, and the purities are nearly 96.8%, 98.1% and 97.8%, respectively. Eluted fractions were analyzed by immunoblotting (Figure S1). The endotoxin levels were measured and found to be below the detection limit (0.25 endotoxin unit (EU)/ml) for purified recombinant proteins at the concentration used in the activation assays (Table S1 Table S2).

### Animal and Treatments

Twenty-four female NZB/W F1 mice at week 6 were purchased from Jackson Laboratories (Bar Harbor, ME, USA) and housed under supervision of the Institutional Animal Care and Use Committee at Chung Shan Medical University, Taichung, Taiwan. The animals were kept under a 12-h light-dark cycle and ambient temperature was maintained at 25°C. Animals were free access to water and standard laboratory chow (Lab Diet 5001; PMI Nutrition International Inc., Brentwood, MO, USA). Animal welfare and experimental procedures were performed according to the NIH Guide for the Care and Use of Laboratory Animals. All the animals at the age of 8 weeks were randomly divided into 4 equal groups (6 mice each group) and injected subcutaneously with 20****ug purified B19-NS1, B19-VP1u, B19-VP2 recombinant proteins or phosphate-buffered saline (PBS) mixed 1:1 (v/v) with Freund's complete adjuvant (Sigma-Aldrich, UK), respectively. The mice were boosted with the same dose mixed 1:1 (v/v) with Freund's incomplete adjuvant (Sigma-Aldrich, UK) twice a week for 3 times and then sacrificed at the age of 16 weeks by CO_2_ asphyxiation. The heart blood and liver tissues were collected and stored at −80°C until use. The reactivity of anti-sera to dsDNA and B19 viral proteins was examined (Table S2).

### ELISA

Direct antigen-specific ELISA kits were used to detect mouse anti-dsDNA total Ig antibodies (Alpha Diagnostic Intl. Inc., TX, USA) according to the manufacture's description. All serum samples were assayed at a dilution of 1/100. After incubation at room temperature for 60 minutes, liquid of each sample well was removed and washed for four times, and subsequently incubated with anti-mouse Ig-horseradish peroxidase conjugated with HRP at a dilution of 1/100. After incubation at room temperature for 30 min, liquid of each sample well was removed, washed and subsequently incubated with the color reaction TMB Chromogen in the dark for 15 min at room temperature. The 100 µl of stop solution was then added to each well and the absorbance (OD) was read at 450 nm within 1 h. For detecting the reactivity of various anti-sera against B19-NS1, VP1u and VP2, 10 mmol/l of recombinant proteins were immobilized on the surface of each sample well of 96-well plates. All anti-sera were assayed at a dilution of 1/1000. The peroxidase conjugated goat anti-mouse IgG (Sigma, Saint Louis Mo, USA) was assayed at a dilution of 1/1000. The color reaction was done with 1 mg/ml substrate ABTs [2, 2'azino-di-(3- ethylbenzthiazolin-6-sulphonic acid)] (Sigma) in the presence of 0.005% H_2_O_2_ at room temperature for 15 min.

### Hematoxylin-eosin Staining

The liver samples of animals were excised and soaked in formalin and covered with wax. Slides were prepared by deparaffinization and dehydration. They were passed through a series of graded alcohols (100%, 95% and 75%), 15 min of each. The slides were then dyed with hematoxylin. After gently rinsing with water, each slide was then soaked with 85% alcohol, 100% alcohol I and II for 15 min each. At the end, they were soaked with Xylene I and Xylene II. Photomicrographs were obtained using Zeiss Axiophot microscopes.

### Preparation of Tissue Extract and Determination of Protein

All procedures were performed at 4°C in a cold room. The tissue samples obtained from NZB/W F1 mice were homogenized in 600 µl PRO-PREP™ solution (iNtRON Biotech, Korea) by 30 strokes using a Dounce Homogenizer (Knotes Glass, Vineland, NJ). The homogenates were centrifuged at 13,000 rpm for 10 min at 4°C and the supernatant was then stored at −80°C until use. Protein concentration of tissue extracts was determined according to the method described elsewhere [20] using bovine serum albumin as standards.

### Immunoblotting

Protein samples were separated in 10% or 12.5% SDS-PAGE and electrophoretically transferred to nitrocellulose membrane (Amersham Biosciences, Piscataway, NJ, USA) described elsewhere [21]. After blocking with 5% non-fat dry milk in (PBS), antibodies against TNF-α receptor, TNF-α, iNOS, COX-2, MMP-9, uPA, IKK-α, IκB, NF-κB (p65) (Santa Cruz Biotechnology, CA, USA) and β-actin (Upstates, Charlottesville, VA, USA) were diluted in PBS with 2.5% BSA and incubated for 1.5 h with gentle agitation at room temperature. The membranes were washed twice with PBS-Tween for 1 h and secondary antibody conjugated with horseradish peroxidase (HRP) (Santa Cruz Biotechnology, Santa Cruz, CA, USA) was added. Pierce's Supersignal West Dura HRP Detection Kit (Pierce Biotechnology Inc., Rockford, IL) was used to detect antigen–antibody complexes. Quantified results were performed by densitometry (Appraise, Beckman-Coulter, Brea, CA, USA).

### Statistical Analysis

All of the statistical analyses were performed using SPSS 10.0 software (SPSS Inc., Chicago, IL). Three independent experiments were repeated. Statistical analyses were performed using the analysis of variance plus posterior multiple comparison test to test the difference. P<0.05 was considered statistically significant. The significant differences were stressed with symbols as shown in figures.

## Results

### Increased Expression of iNOS and COX-2 Proteins in NZB/W F1 Mice Receiving B19-NS1 Protein

**Figure 1:**
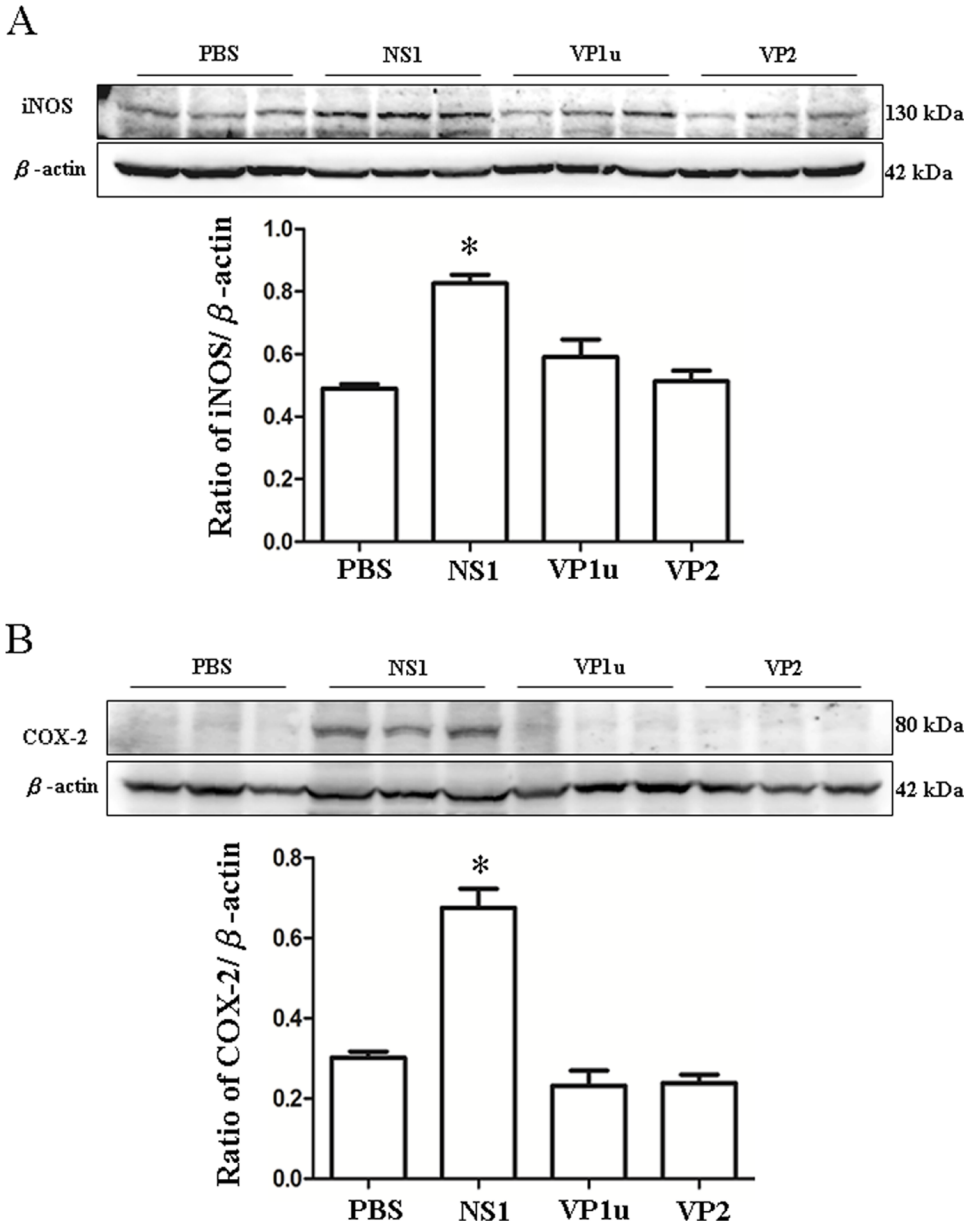
Expression of iNOS and COX-2. Liver lysates obtained from the NZB/W F1 mice receiving PBS, NS1, VP1u or VP2 were probed with antibodies against (A) iNOS and (B) COX-2. Bars represent the relative protein quantification of (A) iNOS and (B) COX-2 on the basis of β-actin. Similar results were observed in three independent experiments, and * indicates the significant difference, P<0.05.

Human parvovirus B19 has been strongly associated with the pathogenesis of SLE. To investigate the influences of B19 viral proteins on livers in SLE, various inflammatory associated proteins including iNOS and COX-2 were examined. Significantly increased iNOS protein level was detected in livers from NZB/W F1 mice receiving B19-NS1 as compared to those mice receiving PBS (Fig. 1A). In contrast, no significant variation on iNOS expression in livers from NZB/W F1 receiving B19-VP1u or VP2 was observed as compared to those mice receiving PBS (Fig. 1A). Additionally, significant increase of COX-2 protein was also detected in livers from NZB/W F1 mice receiving B19-NS1 whereas no significant variation on COX-2 expression in livers from NZB/W F1 receiving B19-VP1u or VP2 was observed as compared to those mice receiving PBS (Fig. 1B). Quantified results were shown in the lower panel of figure 1A and 1B.

### Hepatic Architecture Changes in NZB/W F1 Mice Receiving B19-NS1 Protein

**Figure 2:**
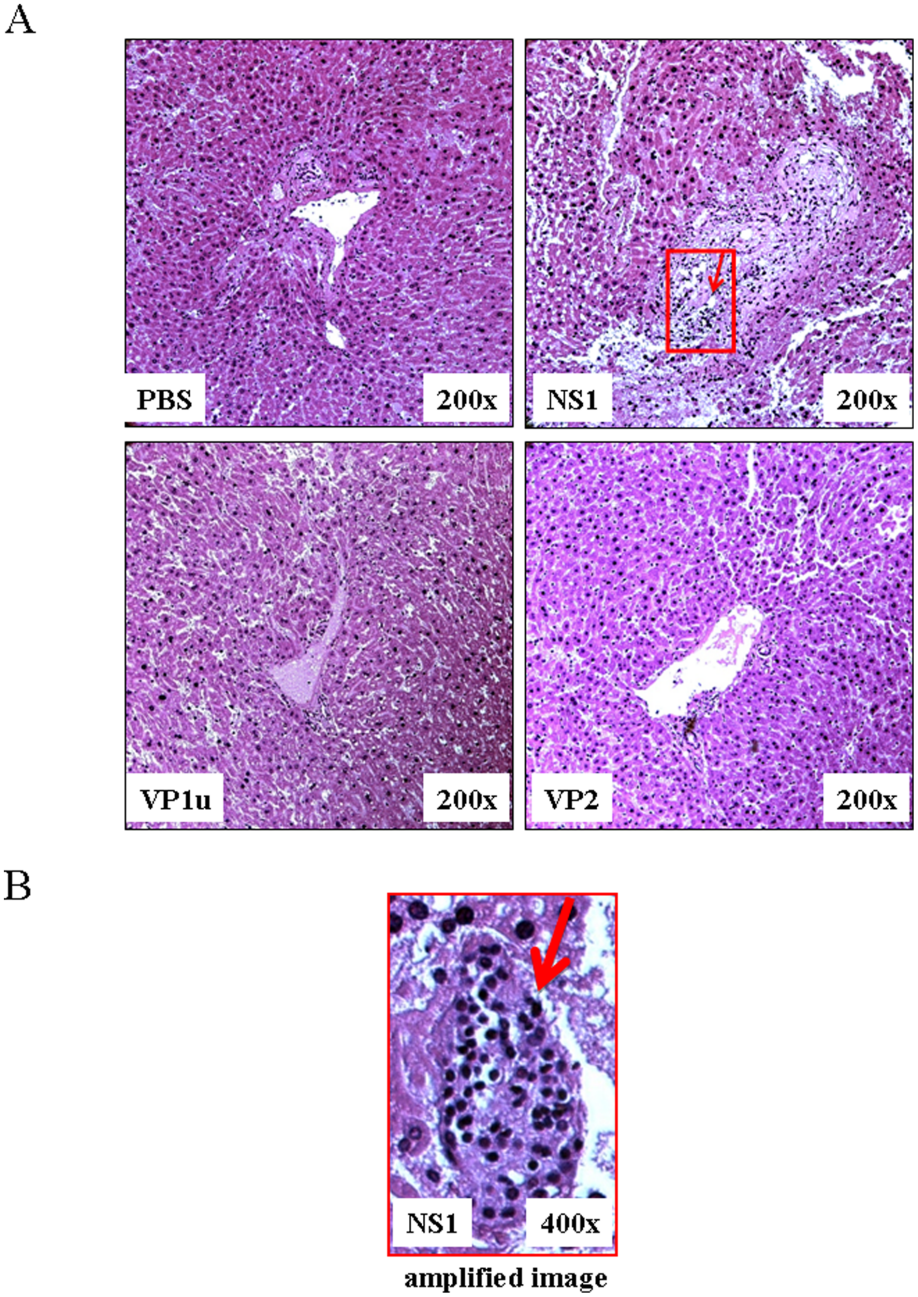
Hepatic histopathological changes in NZB/W F1 mice. (A) Histopathological analysis of hepatic tissue sections with hematoxylin and eosin staining from NZB/W F1 mice receiving PBS, NS1, VP1u or VP2, respectively. These images of hepatic architecture were magnified by 200 times. (B) Amplified image of hepatic tissue sections with hematoxylin and eosin staining from NZB/W F1 mice receiving B19-NS1. The lymphocyte infiltration was indicated by an arrow.

To observe the effects of B19-NS1 protein on hepatic architectures in NZB/W F1 mice, we performed a histopathological analysis on liver tissue stained with hematoxylin and eosin (Fig. 2). More abnormal hepatic architecture and increased interstitial space were observed in livers from NZB/W F1 mice receiving B19-NS1 as compared to those mice receiving PBS, B19-VP1u or VP2, respectively (Fig. 2A). Additionally, markedly lymphocyte infiltration was observed in livers from NZB/W F1 mice receiving B19-NS1 protein as compared to those mice from other groups (Fig. 2B).

### Increased Expression of TNF-α and TNF-α Receptor in NZB/W F1 Mice Receiving B19-NS1 Protein

**Figure 3:**
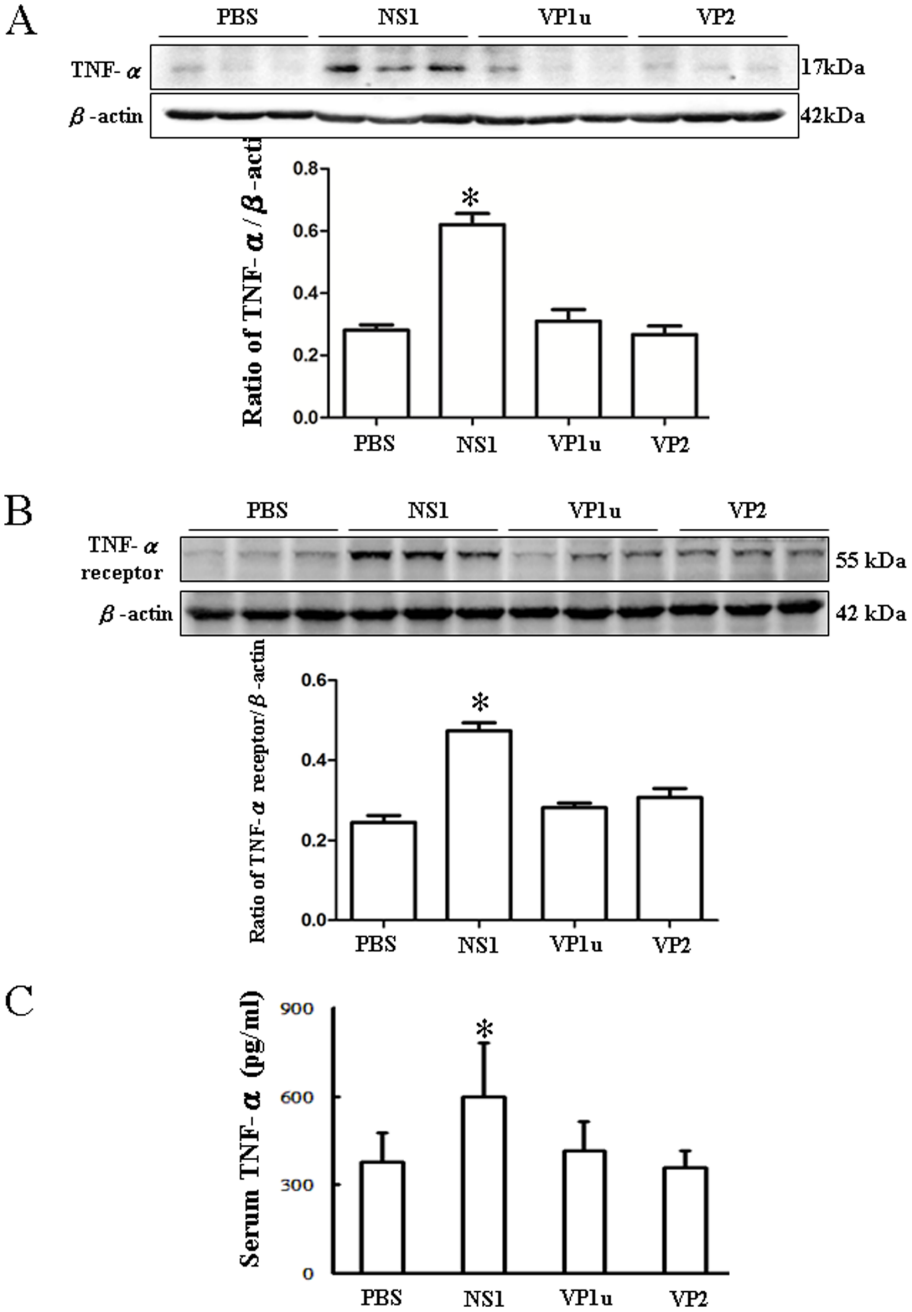
Expression of TNF-α and TNF-α receptor. Liver lysates obtained from the NZB/W F1 mice receiving PBS, NS1, VP1u or VP2 were probed with antibodies against (A) TNF-α and (B) TNF-α receptor. Bars represent the relative protein quantification of (A) TNF-α and (B) TNF-α receptor on the basis of β-actin. (C) Serum TNF-αwas determined with ELISA kit as described in materials and methods (S1). Similar results were observed in three independent experiments, and * indicates the significant difference, P<0.05.

To clarify the possible signaling involved in the B19-NS1 aggravated liver injury in NZB/W F1 mice, the expressions of TNF-α and TNF-α receptor were examined. Significant increase of TNF-α was detected in livers from NZB/W F1 mice receiving B19-NS1 as compared to those mice receiving PBS (Fig. 3A). Consequently, significant increase of TNF-α receptor was also detected in livers from NZB/W F1 mice receiving B19-NS1 as compared to those mice receiving PBS (Fig. 3B). In contrast, no significant variation on both TNF-α and TNF-α receptor expression were observed in livers from NZB/W F1 mice receiving B19-VP1u or VP2 as compared to those mice receiving PBS (Fig. 3A and 3B). Meanwhile, significantly increased serum TNF-α level was also detected in NZB/W F1 mice receiving B19-NS1 as compared to those mice receiving PBS (Fig. 3C). Quantified results were shown in the lower panels of figure 3A and 3B.

### Increased Expression of IKK-α, IκB and NF-κB (p65) in NZB/W F1 Mice Receiving B19-NS1 Protein

**Figure 4:**
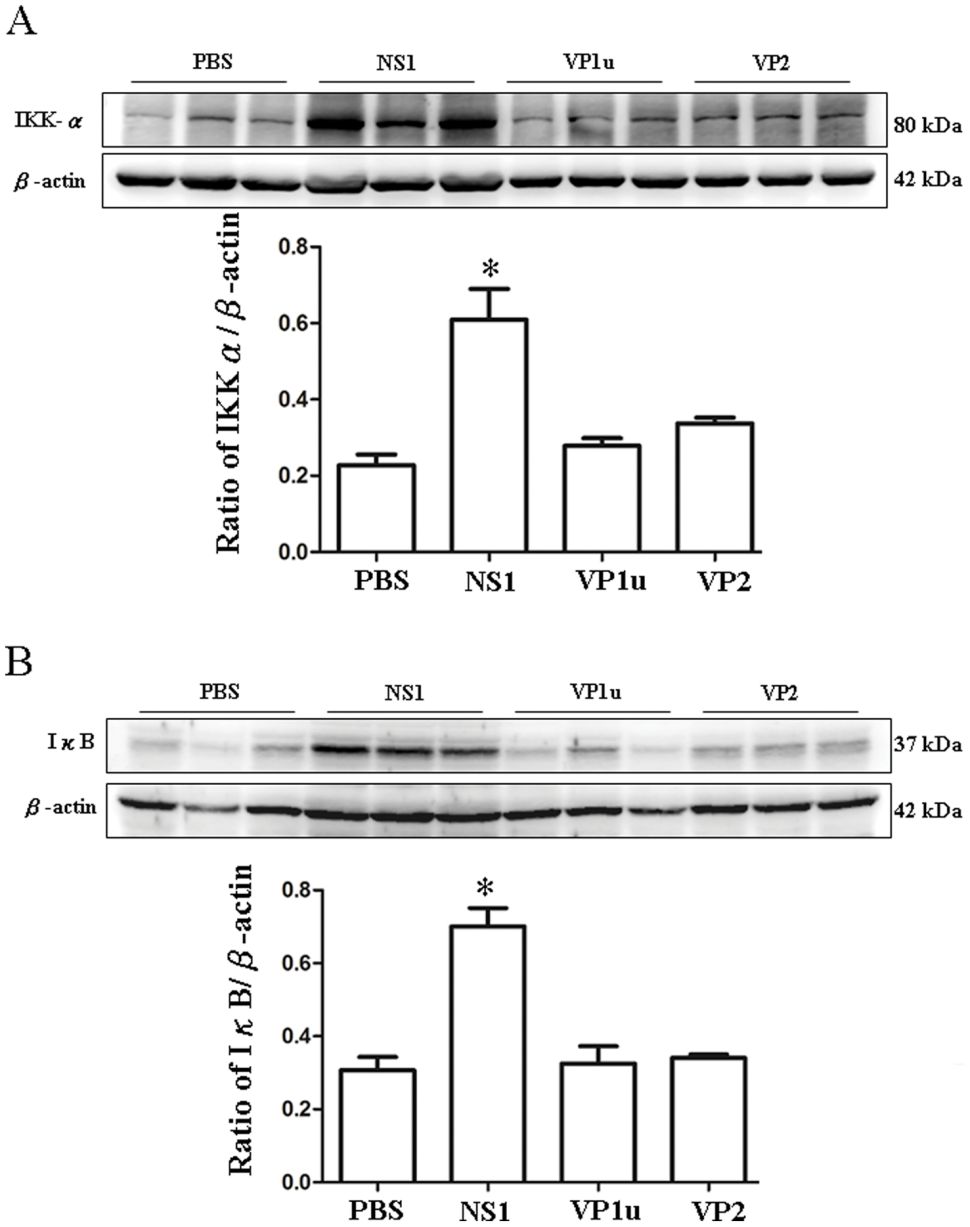
Expression of IKK-α and IκB. Liver lysates obtained from the NZB/W F1 mice receiving PBS, NS1, VP1u or VP2 were probed with antibodies against (A) IKK-α and (B) IκB. Bars represent the relative protein quantification of (A) IKK-α and (B) IκB on the basis of β-actin. Similar results were observed in three independent experiments, and * indicates the significant difference, P<0.05.

**Figure 5:**
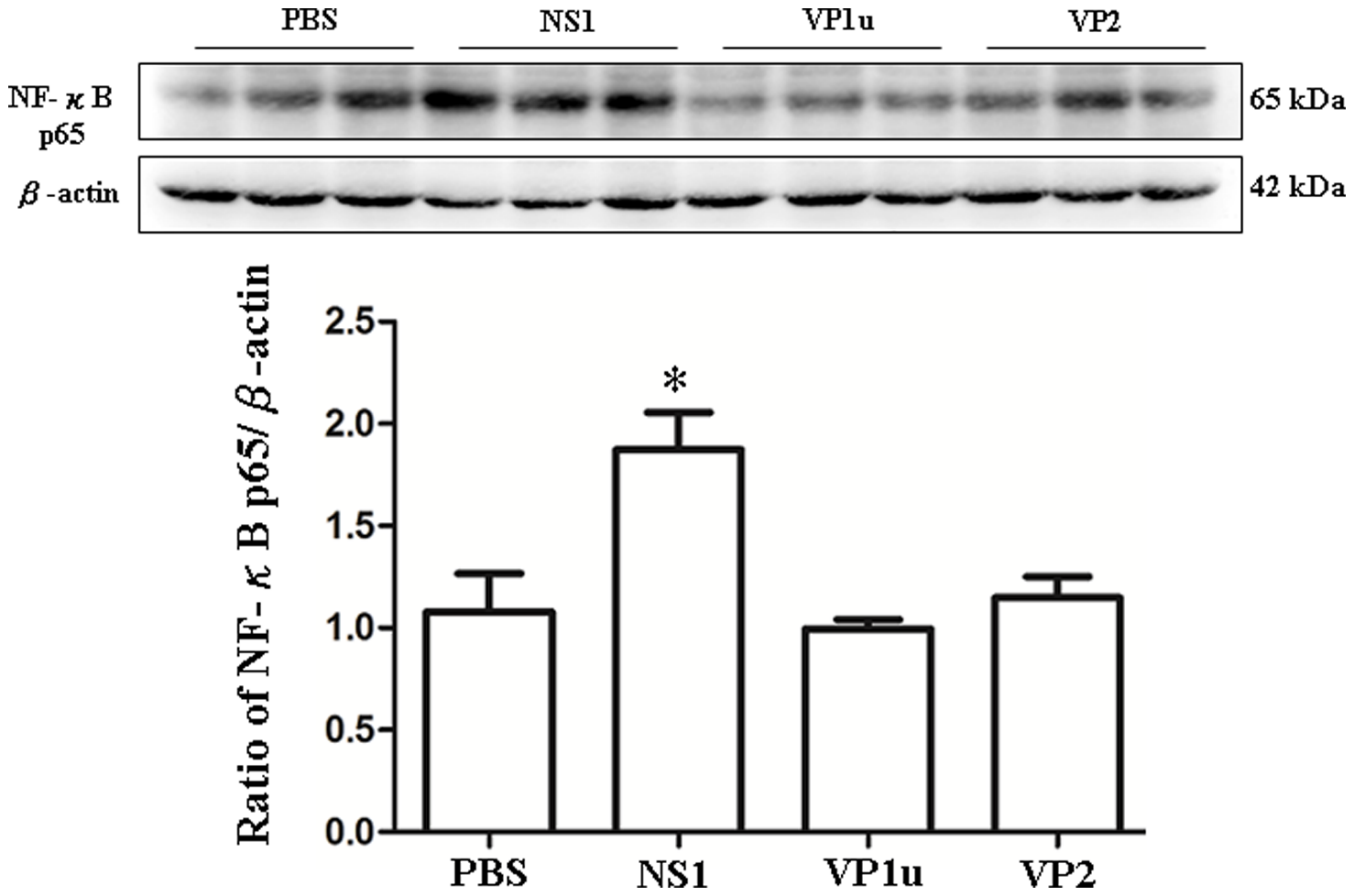
Expression of NF-κB p65. Liver lysates obtained from the NZB/W F1 mice receiving PBS, NS1, VP1u or VP2 were probed with antibodies against NF-κB p65 Bars represent the relative protein quantification of NF-κB p65 on the basis of β-actin. Similar results were observed in three independent experiments, and * indicates the significant difference, P<0.05.

To further investigate the signaling molecules involved in the B19-NS1 enhanced TNF-α expression, the downstream molecules of TNF-α including IKK-α, IκB, NF-κB (p65) were examined. Significant increases of IKK-αand IκB were detected in livers from NZB/W F1 mice receiving B19-NS1 as compared to those mice receiving PBS (Fig. 4A and 4B). In contrast, no significant variation on IKK-αand IκB expression were observed in livers from NZB/W F1 mice receiving B19-VP1u or VP2 as compared to those mice receiving PBS (Fig. 4A and 4B). Quantified results were shown in the lower panels of figure 4A and 4B. Moreover, significant increase of NF-κB (p65) was also detected in livers from NZB/W F1 mice receiving B19-NS1 as compared to those mice receiving PBS whereas no significant variation on NF-κB (p65) level was observed in NZB/W F1 mice receiving B19-VP1 u or VP2 as compared to those mice receiving PBS (Fig. 5). Quantified results were shown in the lower panel of figure 5.

### Increased MMP-9 and uPA Expression in NZB/W F1 Mice Receiving B19-NS1 Protein

**Figure 6:**
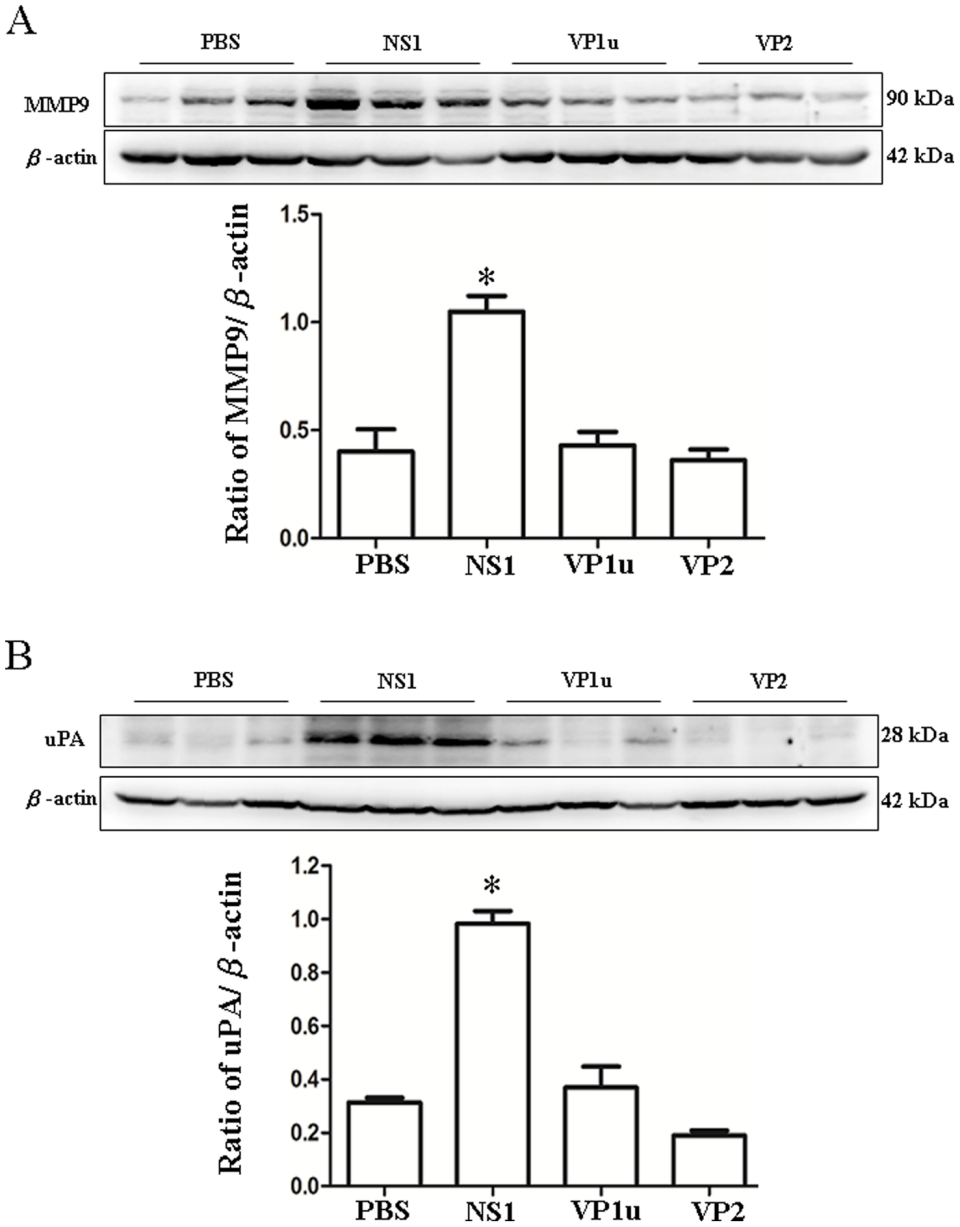
Expression of MMP9 and uPA. Liver lysates obtained from the NZB/W F1 mice receiving PBS, NS1, VP1u or VP2 were probed with antibodies against (A) MMP9 and (B) uPA. Bars represent the relative protein quantification of (A) MMP9 and (B) uPA on the basis of β-actin. Similar results were observed in three independent experiments, and * indicates the significant difference, P<0.05.

MMP-9, a consequent molecules in TNF-α signaling, is known as an indicator playing important roles in hepatic disorders. To further examine the effect of B19-NS1 protein on MMP-9 expression, Immunoblots were preformed to examine the expression of MMP-9. Significant increase of MMP-9 was detected in livers from NZB/W F1 mice receiving B19-NS1 as compared to those mice receiving PBS (Fig. 6A). Additionally, the expression of uPA protein, an upstream activator of MMP-9, was also examined. As shown in figure 6B, significant increase of uPA protein was observed in liver from NZB/W F1 mice receiving B19-NS1 as compared to those mice receiving PBS (Fig. 6B). In contrast, no significant variation on both MMP9 and uPA expression were observed in livers from NZB/W F1 mice receiving B19-VP1u or VP2 as compared to those mice receiving PBS (Fig. 6A and 6B). Quantified results were shown in the lower panels of figure 6A and 6B.

## Discussion

Although B19 infection has been implicated in pathogenesis of liver diseases and development of SLE, the effects of B19 and its viral proteins including NS1, VP1u and VP2 on hepatic injury in SLE is still obscure [6], [9]–[16]. In the present study, we revealed the aggravated effects of B19 NS1 protein on hepatic injury in NZB/W F1 mice by significantly enhancing the expressions of iNOS and COX-2 proteins and lymphocyte infiltration. Additionally, significant increase of MMP-9 through TNF-α/NF-κB (p65) signaling was also detected.

Elevated inducible nitric oxide synthase (iNOS) has been reported in patients with SLE [22]. According to a recent clinical research of 72 SLE patients, significant higher oxidative stress including iNOS level has been associated with the SLE Disease Activity Index (SLEDAI) and recognized to the pathogenesis of SLE [23]. Similar result was also observed in lupus-prone mice [24]. Accordingly, cyclooxygenase type 2 (COX-2) is also known to play pivotal roles in development of inflammatory diseases and associated with the pathogenesis of SLE [25]–[26]. A previous study has demonstrated that some COX-2 inhibitors are able to suppress the production of pathogenic autoantibodies to DNA by causing autoimmune T-cell apoptosis [27]. Similar result was also demonstrated in a lupus-prone murine model [28]. These studies did stress the roles of iNOS and COX-2 in development of SLE.

In the current study, significant increases of iNOS and COX-2 expression were detected in livers from NZB/W F1 mice receiving B19-NS1 but not VP1u or VP2 as compared to those mice receiving PBS. By the way, markedly lymphocyte infiltration was observed in NZB/W F1 mice receiving B19-NS1. These findings suggested the effect of B19-NS1 but not VP1u or VP2 on aggravating the hepatic injury in SLE by enhancing the expression of iNOS and COX-2.

**Figure 7:**
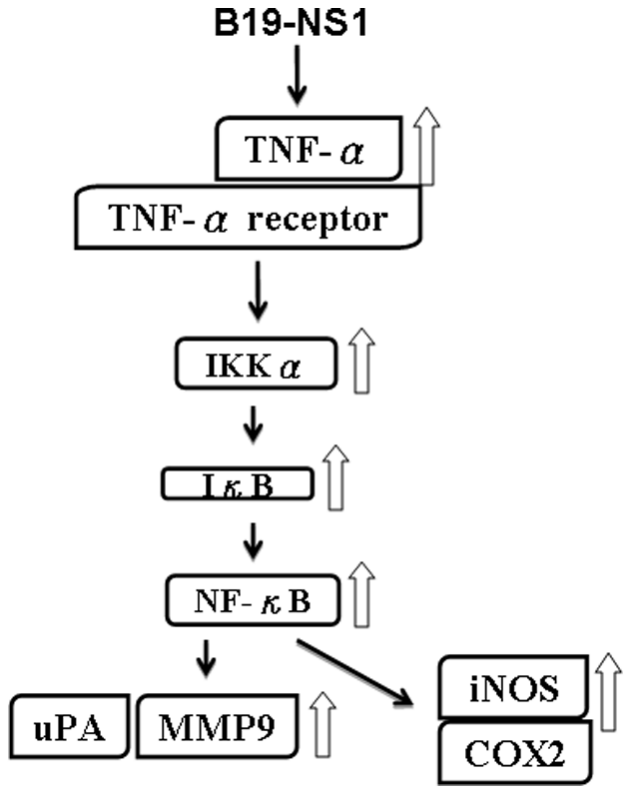
Schematic illustration of the molecular mechanisms involved in the B19 NS1-induced hepatic inflammation in NZB/W F1 mice. B19-NS1 increases the expression of TNF-α and its receptor, which elicit late activation of IKK-αand IκB. Consequently, NF-κB is also activated to induce the expressions of inflammatory associated molecules, uPA and MMP9. The other downstream inflammatory-realted molecules of TNF-α, including iNOS and COX, are also markedly indued.

Matrix metalloproteinase-9 (MMP-9) has been postulated with the pathogenesis of autoimmune diseases including SLE [29]. Various studies have reported that elevated MMP-9 activity plays crucial role in development of SLE in both human and lupus-prone mice [30]–[31]. Indeed, the cleavage of myelin basic protein or type II gelatins by MMP-9 will generate remnant epitopes and contribute to develop autoimmunity [32]. Additionally, TNF-α is known to be involved in the signal pathway of MMP-9 induction, which degrades extracellular matrix in the inflammatory responses [33]. Accordingly, IKK and IKB, the downstream molecules for TNF-α induced MMP-9 expression, are known to be activated prior to the transcription of MMP-9 promoter through NF-κB sites [34]–[35]. Consistently, our experimental results revealed that B19-NS1 aggravatedly induces the expression of MMP-9 in livers of NZB/W F1 mice via TNF-α signaling, which also elicits the activation of IKK-α, IκB and NF-κB (Fig. 7).

B19 NS1 is known as a cytotoxic protein with multi-function. Besides the ATPase and DNA helicase activity [36], B19 NS1 protein has been described as a transactivator of the viral p6- as well as a variety of cellular promoters. These include the promoter region controlling the expression of TNF-α and IL-6 genes [37]–[38]. Elevated levels of TNF-α have been shown to be present in patients during the acute and convalescent phases of B19 infection [39]. The prolonged or continuous presence of these proinflammatory cytokines during acute-convalescent and persistent B19 infection, respectively, may result in the induction of long lasting clinical symptoms and autoimmune reactions [6]. Although causality is often difficult to conclude, these studies did imply that B19 NS1 is possibly involved in the induction of autoimmunity and can’t be negated [6], [39]. Briefly, NZB/W F1 is a well known spontaneously lupus mice model [40], which has been used for investigating SLE for several decades.

Therefore, we simply intend to clarify the influences of B19 NS1 proteins in NZB/W F1 mice and firstly report the aggravated effects of B19 NS1 on liver injury in NZB/W F1 mice. Since B19-infection is commonly detected in patients with SLE [41], these findings may provide a possible explanation on the effects of B19 NS1 in SLE patients with B19-infection.

## Supporting Information

Figure S1 **SDS-PAGE (A) and Immunoblottings (B) of purified recombinant B19-NS1 (77 kDa), -VP1u (47 kDa) and -VP2 (58 kDa) proteins probed with rabbit anti-B19-NS1 IgG, rabbit anti-B19 VP1u IgG and mouse anti-B19-VP2 IgG, respectively.**
(TIF)

Table S1 **Endotoxin assay for different preparations of B19 viral proteins.**
(DOC)

Table S2 **Reactivity of anti-sera to dsDNA and B19 viral proteins.**
(DOC)
