# Supplementary figures and images for: Human Parvovirus B19 NS1 Protein Aggravates Liver Injury in NZB/W F1 Mice
Source: PLoS One. 2013 Mar 21;8(3):e59724. doi: 10.1371/journal.pone.0059724 (PMC3605340; doi:10.1371/journal.pone.0059724)

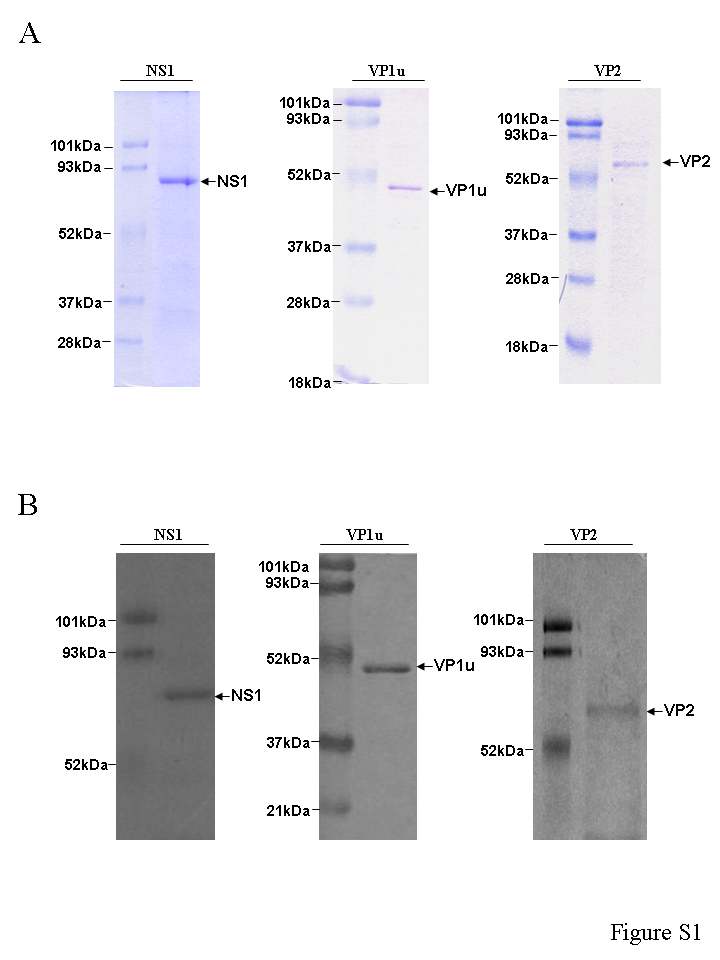

Supplement: Figure S1 — SDS-PAGE (A) and Immunoblottings (B) of purified recombinant B19-NS1 (77 kDa), -VP1u (47 kDa) and -VP2 (58 kDa) proteins probed with rabbit anti-B19-NS1 IgG, rabbit anti-B19 VP1u IgG and mouse anti-B19-VP2 IgG, respectively. (TIF) [file pone.0059724.s001.tif]
