# Supplementary material for: Human Parvovirus B19 NS1 Protein Aggravates Liver Injury in NZB/W F1 Mice
Source: PLoS One. 2013 Mar 21;8(3):e59724. doi: 10.1371/journal.pone.0059724 (PMC3605340; doi:10.1371/journal.pone.0059724)
Supplement: Table S1 — Endotoxin assay for different preparations of B19 viral proteins. (DOC) [file pone.0059724.s002.doc]

**Table S1. Endotoxin assay for different preparations of B19 viral proteins**

| Preparation of B19 viral proteins | Endotoxin units (EU)/ml^a^ |
| --- | --- |
| NS1* | 0.12 |
| VP1u* | 0.06 |
| VP2* | 0.06 |

The value of EU/ml ≥ 0.25 will be considered as positive for endotoxin.

^a^ Endotoxin Standard is positive at 0.06 EU/ml and negative at 0.03 EU/ml.

* Indicates the original concentration is 100μg/ml.
