# Supplementary material for: Human Parvovirus B19 NS1 Protein Aggravates Liver Injury in NZB/W F1 Mice
Source: PLoS One. 2013 Mar 21;8(3):e59724. doi: 10.1371/journal.pone.0059724 (PMC3605340; doi:10.1371/journal.pone.0059724)
Supplement: Table S2 — Reactivity of anti-sera to dsDNA and B19 viral proteins. (DOC) [file pone.0059724.s003.doc]

**Table S2. Reactivity of anti-sera to dsDNA and B19 viral proteins**

|  |  |  | Anti-sera | |  |
| --- | --- | --- | --- | --- | --- |
| Antigens | PBS (n=6) | NS1 (n=6) | | VP1u (n=6) | VP2 (n=6) |
| dsDNA | 1.789±0.678 | 2.089±0.531 | | 1.969±0.716 | 1.897±0.601 |
| NS1 | 0.171±0.036 | 2.868±0.569^a^ | | 0.213±0.086 | 0.286±0.105 |
| VP1u | 0.199±0.087 | 0.301±0.126 | | 2.749±0.706^a^ | 0.275±0.096 |
| VP2 | 0.163±0.069 | 0.353±0.107 | | 0.261±0.111 | 2.367±0.697^a^ |

^a^ indicates significant differences (*P*<0.05) as compared with the value of PBS.

all values are optical density and presented as mean±SD.
